# Multilevel factors associated with clinical breast examination uptake among women in the Northern Region of Ghana: a cross-sectional survey

**DOI:** 10.1186/s12885-025-14550-w

**Published:** 2025-10-01

**Authors:** Agani Afaya, Hyeonkyeong Lee, So Yoon Kim, Chang Gi Park, Min Kyeong Jang, Sue Kim

**Affiliations:** 1 Department of Nursing, School of Nursing and Midwifery, University of Health and Allied Sciences, Ho, Ghana; 2 College of Nursing, Mo-Im Kim Nursing Research Institute, Yonsei University, Seoul, Korea; 3 Asian Institute for Bioethics and Health Law, Department of Medical Humanities and Social Sciences, College of Medicine, Yonsei University, Seoul, Korea; 4 Department of Population Nursing Science, College of Nursing, University of Illinois, Chicago, IL USA

**Keywords:** Breast cancer, Clinical breast examination, Multilevel factors

## Abstract

**Background:**

Despite the World Health Organization’s recommendation of clinical breast examination (CBE) in resource-limited settings where mammography services are unavailable, the participation rate among women in Ghana remains low. This study examined multilevel factors associated with CBE uptake among women in the Northern Region of Ghana.

**Methods:**

A multi-stage cluster sampling technique was employed to recruit women aged 25 years and older from 30 communities in the Tamale metropolis, Northern Region of Ghana. To determine the factors associated with CBE uptake, multilevel logistic regression models were fitted. The outcome of the fixed effects analysis was presented using adjusted odds ratios (aOR), with a 95% confidence interval (CI) and a p-value of less than 0.05.

**Results:**

Approximately 1,543 women were recruited from 30 communities with 48 clusters. The prevalence of CBE uptake was 23%. From the fixed effects results, women with tertiary education (aOR = 2.53, 95%CI = 1.49–4.29) were more likely to have CBE compared to those with no formal education. Women with increased knowledge of breast cancer (BC) were more likely to undergo CBE (aOR = 1.04, 95%CI = 1.00-1.07). The higher the perceived susceptibility to BC, the higher the likelihood of undergoing CBE (aOR = 1.25, 95%CI = 1.06–1.47). Women with increased perceived barriers to BC screening (aOR = 0.54, 95%CI = 0.41–0.73) and higher perceived fear of BC (aOR = 0.72, 95%CI = 0.57–0.91) were less likely to undergo CBE. Women who received recommendations from friends (aOR = 1.99, 95%CI = 1.25–3.18) and healthcare providers (aOR = 1.55, 95%CI = 1.00-2.39) were more likely to utilize CBE compared to their counterparts. Women in communities with health facilities (aOR = 2.14, 95%CI = 1.43–3.21) and those who resided in urban areas (aOR = 1.72, 95%CI = 1.10–2.69) were more likely to utilize CBE than their counterparts.

**Conclusion:**

The findings signify that a multifactorial systematic approach is required to increase CBE uptake among Ghanaian women. We recommend a comprehensive community-based education program about BC, targeted at raising awareness of breast health and improving the knowledge of the disease, together with the benefits of CBE uptake. Concurrently, improving access to health facilities and CBE services is needed.

**Supplementary Information:**

The online version contains supplementary material available at 10.1186/s12885-025-14550-w.

## Background

Breast cancer (BC) is the most diagnosed cancer across the globe and the leading cause of cancer mortality among women [1]. As of 2020, it was reported that nearly 2.3 million women had BC, representing 11.7% of all cancer cases in the world [1]. Evidence suggests that BC cases with disproportionately high mortality rates are increasing significantly in low-resource countries [1]. Despite evidence from literature that the survival rate of BC is rising in most high-income countries, sub-Saharan Africa (SSA) has the lowest survival rate internationally [2, 3]. For instance, the five-year survival rate in SSA is practically at or below 50%, which means that for every two women diagnosed with BC, one will die within five years after diagnosis [4]. Ghana’s scenario is comparable because BC is currently the most common malignancy and the main reason for cancer-related death in women. According to research evidence, approximately 30% of BC cases in Ghana are diagnosed in women under the age of 35, making it a public health concern [5]. This current trend in BC diagnosis implies a relative shift in the burden of BC to younger women in their thirties when compared to high-income or Western countries [6].

It has been established that BC screening increases the likelihood of early detection and treatment, lowers mortality, and increases the survival rates of women [7]. Clinical breast examination (CBE) is recommended for low-resource settings where mammography screening services are limited. It has been established that CBE ensures early BC detection, evidenced by increased detection of < 2 cm diameter BC and approximately 40% more detections with stage one or two of the disease [8]. A two-decade prospective, clustered randomized controlled trial study in Mumbai, India, found that biennial CBE among women of all ages resulted in considerable BC downstaging [9]. Mittra and colleagues from Mumbai, India, discovered a non-significant 15% reduction in BC mortality in the intervention group compared to the control group [9].

Despite clinical evidence that this screening modality improves early diagnosis and reduces BC-related mortality, participation rates among women in Ghana remain low [10]. Due to the poor screening rates, most Ghanaian women present to health facilities with advanced stages of BC (stages III & IV), resulting in low survival rates [11]. According to a study conducted in the Ashanti region of Ghana, around 52.35% of women were diagnosed with advanced BC, with a 47.9% five-year survival rate [11]. Women diagnosed with advanced-stage BC have a low survival rate, which has been attributed to late presentation to health facilities, a lack of knowledge, and poor BC screening practices in Ghana [10, 11]. Most studies conducted in Ghana have focused on individual factors impacting women’s screening behavior [10]. Lack of screening programs, cultural attitudes, lack of health insurance coverage, cost of treatment, and low socioeconomic position have all been connected to the low BC screening in Ghana [10]. To date, there are limited studies that have examined the multiple levels of factors that are associated with CBE uptake in Ghana, particularly in the Northern region of Ghana. At the time of this study, the whole Northern region of Ghana lacked a mammography screening center, and CBE was only available in secondary and tertiary-level health facilities. Investigating multilevel factors could thus serve as the foundation for developing public health initiatives, interventions, and policies concentrating on the combination of factors to increase women’s screening behavior. This study was based on the premises of the Ecological Model, which considers multilevel factors that influence the health behavior of individuals in an ecological system [12]. The Ecological Model stretches beyond the individual level to explain how other factors affect health behavior. Therefore, it is imperative to assess multilevel factors using the Ecological Model to derive interventions and policies that target multifaceted factors influencing CBE uptake among women in Ghanaian communities.

## Methods

### Study design

This study employed a descriptive cross-sectional design to examine the relational effects of multilevel factors influencing CBE uptake among women. This study adhered to the STROBE reporting guidelines (https://www.strobe-statement.org/).

### Study setting

The study was conducted in the Tamale Metropolis, in the Northern region of Ghana. The Tamale metropolis has three sub-metropolises that make up the Metropolitan Assembly, namely: Tamale North, Tamale South, and Tamale Central. The metropolis has a population of 374,744 people, with 185,051 men and 189,693 women, as per the Ghana 2021 population and housing census. There are 117 communities in the Metropolitan Assembly, including urban [41 (35%)], peri-urban [15 (13%)], and rural [61 (52%)]. In the Tamale Metropolis, there were 89,011 households and 9,234 non-household residents. There is a tertiary-level hospital that acts as a referral hub for all of Ghana’s northern regions. The city is also home to three secondary-level medical facilities. The tertiary-level facility contains a cancer unit that treats both outpatients and inpatients. The three additional secondary-level hospitals offer CBE services in outpatient departments but do not have a BC unit.

### Inclusion and exclusion criteria

This study included Ghanaian women who resided within the Tamale metropolis and were aged between 25 and 75 years and had no history of BC. The exclusion criteria involved women who were mentally challenged or self-reported any form of breast disease.

### Sampling technique

A multi-stage cluster sampling method, i.e., a stratified two-stage cluster sampling technique, was used as follows. First Primary Sample Units (PSUs) or clusters within communities that included both urban and rural areas were selected by systematic random sampling. Next, households within the chosen clusters were identified via a systematic random sampling procedure. The overall number of women chosen from each cluster was proportional to its population based on the demarcation by the Ghana Demographic and Health Survey (GDHS) clusters.

Using the GDHS mapping of the enumeration areas, 33 women were selected from each cluster. In determining the sample size, a 38% CBE rate among women based on a previous study in the Northern region of Ghana [13], an Intra-Class Coefficient (ICC) of 0.1 and a cluster size of 30 based on GDHS, the study required a sample size of 1,412 for estimating the expected proportion with 5% absolute precision and 95% confidence. Considering a 10% non-response rate [14], the sample size was adjusted to 1553. Thus, we selected 48 clusters (30 rural, 18 urban) via a random number generation function in Microsoft Excel for the three-sub metropolitan assemblies, and about 33 women were randomly selected per cluster. After excluding 10 incomplete data points, data from 1,543 women were used for analysis.

### Measurements

#### Outcome measures

The main outcome variable was CBE uptake within the past year, through self-report to the question “In the past year, have you had your breast examined by a health professional to screen for cancer”? The response options were binary (yes/no).

#### Independent variables

Intrapersonal, interpersonal, and contextual variables were measured as follows.

#### Intrapersonal factors

##### Breast cancer knowledge

BC knowledge was measured using the Breast Cancer Awareness Measure (BCAM) tool [15], modified and validated among women in Nigeria [16]. The 21-item BCAM measures knowledge in four subdomains: knowledge of risk factors (4 items), knowledge of warning signs (11 items), knowledge of protective factors (3 items), and knowledge of BC best practice (3 items). The knowledge of risk, protective factors, and best practices on BCAM subscales are measured using a five-point Likert scale (1 strongly disagree, to 5 strongly agree). Responses 4 and 5 were coded as zero (0), and the rest were coded as one (1). The subscale on knowledge of warning signs was measured using yes/no/don’t know responses, with only correct answers coded as 1. To the item “Who is most likely to get breast cancer?” the correct response was coded as one (1) and the incorrect response was coded as zero (0). Higher summed total scores (possible range: 0–22) indicated greater knowledge of BC. The Cronbach alpha reported at development [16] were acceptable for the knowledge of risk factors subscale (α = 0.77), warning signs (α = 0.81), and protective factors (α = 0.67). The original researchers did not report the Cronbach alpha value for knowledge on best practice [16]. In this study, internal consistency was good: knowledge of risk factors (α = 0.81), warning signs (α = 0.97), protective factors (α = 0.76), and BC best practice (α = 0.78).

##### Perceived susceptibility, benefits, and barriers

The Champion Health Belief Model Scale (CHBMS) [17]which was revised and validated for assessing BC screening behavior in Malaysia (CHBMS-BC-M) [18]. was used to assess perceived susceptibility, benefits, and barriers. The CHBMS-BC-M version was chosen because it included both CBE and mammograms (MMG) as BC screening, whereas the CHBMS measured only MMG [17]. This 21-item scale comprises perceived susceptibility (3 items), perceived benefits (4 items), and perceived barriers (14 items). A five-point Likert scale (1 strongly disagree, to 5 strongly agree) is used, and high point average scores (possible range: 1–5) from each of the subscale indicated an increased perceived susceptibility and benefits, whereas higher scores for barriers indicated a greater perception of barriers to BC screening. The Cronbach alpha for each of the subscales was originally reported as follows: perceived susceptibility (0.92), benefits (0.59), and barriers (0.78) [18]. The Cronbach alpha values in this study were good: perceived susceptibility α = 0.91), benefits (α = 0.62), and barriers (α = 0.82).

##### Fatalism

The 15-item Powe Fatalism Scale [19] which was modified to 11 items in Ghana [20] was used. For the four subscales: predetermination (5 items), inevitable death (2 items), religiosity (1 item), and pessimism (3 items), responses are recorded as yes (1) or no (0) and summed (possible range: 0–11) with higher scores indicating greater fatalistic belief. Mayo and colleagues (2003) reported a Cronbach’s alpha of 0.88 [20]. In this study, the Kuder-Richardson (KR-20) value was 0.78.

##### Perceived treatment belief, stigma, and fear of breast cancer

This study selected three out of the six subscales (perceived knowledge, perceived treatment belief, perceived need for health check, perceived stigma, perceived fear, and perceived risk) in the Breast Cancer Perception Scale [21], which was developed for use with non-breast cancer women. The three selected subscales included perceived treatment belief (5 items), perceived stigma (4 items), and perceived fear (4 items). Using a five-point Likert scale (1 strongly disagree, to 5 strongly agree), the point-average (range: 1–5) for each subscale was calculated separately, with items 7 and 9 in perceived treatment belief reverse-coded. Higher scores indicated a more positive perceived treatment belief, greater stigma, and greater fear of BC. Cronbach alpha values reported by the developers were good for the perceived treatment belief subscale (0.85), perceived stigma (0.81), and perceived fear (0.89) [21]. In the current study, the Cronbach alpha values were acceptable for perceived treatment belief (0.71), perceived stigma (0.66), and perceived fear (0.93).

##### Cultural belief

The 17-item Cultural Belief Instrument developed by Ferrans et al. [22] was used to measure cultural beliefs that act as barriers to BC screening among women. The four subscales measure cultural beliefs on breast lumps (4 items), self-help techniques (4 items), faith-based beliefs (4 items), and futility of treatment (5 items). Items were measured using True or False responses, and higher summed scores (range: 0–16) indicated greater cultural beliefs that act as barriers to BC screening. Item 16 was not included in the calculation of the total score as recommended by the authors [22]. The Cronbach alpha was 0.73 at development [22] and.71 in this study.

##### Sociodemographic characteristics

Age (in years), educational attainment, marital status, employment status, religious affiliation, level of monthly income, place of residence, ease of access to hospitals and screening facilities, health insurance coverage, cost of medical care, exposure to mass media (TV, radio, newspapers), parity, menopause, and family history of BC were among the sociodemographic characteristics that were derived from the literature.

#### Interpersonal factors

##### Recommendations for CBE

The recommendations for CBE from friends (1 item), families (1 item), and healthcare professionals (1 item) were individually assessed. Response options were yes/no for each of the questions and were analyzed separately.

##### Social support for breast cancer screening

The 19-item Medical Outcome Study (MOS) Social Support Survey [23] was used to examine women’s social support for BC screening. Emotional/informational support (8 items), tangible support (4 items), loving support (3 items), and pleasant social contact (3 items) are the four subscales. The point average score (range: 1–5) was calculated using a five-point Likert (1 never, to 5 always). Higher scores indicated greater social support. Cronbach alpha was greater than 0.91 for all subscales in the original study [23], and it was 0.98 in this study.

#### Contextual factors

The following community-level variables were measured as contextual factors: Availability of hospital/BC screening centers was assessed using a binary response (yes/no). Distance to the health facility was assessed using GPS coordinates and measured in kilometers (km). The place of residence was categorized into binary responses (rural and urban areas). Finally, the geographical location was measured using the three subdivisions of the Tamale metropolis (Tamale Central, Tamale South, and Tamale North).

#### Data collection procedures

Prior to data collection, an organized standardized training program was carried out for the research assistants (RA, *n* = 9) to maintain uniformity in data collection. The RAs were allocated to the various sub-metropolitan assemblies and specific clusters in rural and urban areas. Random visits were made to households within the communities to assess their eligibility for the study. For women who did not respond upon visits, their households were not included. Before the women were recruited, the RAs explained the aim and purpose of the study in line with the instructional sheet, which included the details of the researcher, purpose, benefits, risks of participation, and principles of confidentiality. The participants were also informed about their right to withdraw from the study at any point in time. After checking for eligibility, RAs obtained written and verbal consent and read the survey questions to the participants, and recorded their responses through the Kobo toolbox questionnaire, an online data collection tool (https://www.kobotoolbox.org/). RAs then conducted face-to-face interviews to ensure consistency in data collection. The data collection tool for the study has been attached (Supplementary file 1).

### Statistical analysis

Stata version 16 (StataCorp, LP, College Station, TX, USA) was used to analyze the data. Descriptive statistics were done for the distribution of CBE uptake across the categories of the explanatory variables. The association between the outcome variable (CBE) and the selected explanatory variables was estimated using a chi-square test and an independent t-test. The multivariate multilevel analyses considered the independent variables that were significant in the bivariate analysis. To determine factors associated with CBE uptake, multilevel fixed effects binary logistic regression models were fitted. In total, four models (Model 0, Model I, Model II, and Model III) were built: the first model (Model 0) was fitted as an empty model without predictors (random intercept). The second model (Model I) included intrapersonal-level factors against CBE uptake. Utilizing the interpersonal level components and CBE uptake, the third model (Model II) was fitted. By making adjustments for the contextual level variables, the final model (Model III) was fitted. The outcomes of the fixed effects analysis were presented using adjusted odds ratios (aOR), which had a 95% confidence interval (CI) and a p-value of less than 0.05. According to Merlo and colleagues [24], the Intra-Class Correlation (ICC) was employed to evaluate random effects (measures of variability). The best-fit model was used to present the results, and the log-likelihood ratio test was performed to assess the models’ suitability. The explanatory factors and the outcome measures were examined for potential multicollinearity using a multicollinearity diagnostic test. A Variance Inflation Factor (VIF) of more than 10 was not required for any of the explanatory factors to be excluded.

## Results

### Sociodemographic characteristics of participants

As shown in Table 1, the mean age of the 1,543 participants was 40.38 ± SD = 11.64 years and ranged from 25 to 75 years. The majority were either married or cohabiting (78.3%). Nearly half of the women had no formal education (41.7%), and the majority were Muslims (88.5%). Roughly two-thirds (65.3%) received a monthly income (< 500 Ghanaian Cedis, GHS) less than the daily minimum wage in Ghana. Two-thirds of women resided in rural areas (66.1%) while 56.2% were 5–10 km away from a health facility that offers CBE.

**Table 1:** Participants’ characteristics (*N* = 1,543)

Variables	Categories	Possible Range	*N*(%)	M ± SD
**Intrapersonal factors**				
Age (years)				40.38 ± 11.64 (min 25, max 75)
Marital status	Never married/single		241 (15.6)	
	Divorced/widowed		94 (6.1)	
	Married/cohabitating		1208 (78.3)	
Educational status	No education		644 (41.7)	
	Primary/junior high		392 (25.4)	
	Secondary		317 (20.6)	
	Tertiary		190 (12.3)	
Religious status	No religion/Traditionalist		12 (0.8)	
	Christian		165 (10.7)	
	Muslim		1,366 (88.5)	
Employment status	No		968 (11.1)	
	Yes, Part-time		172 (11.2)	
	Yes, Full time		403 (26.1)	
Monthly income level	Less 500 GHS		1,007 (65.3)	
	500–1000 GHS		345 (22.3)	
	> 1000 GHS		191 (12.4)	
Health insurance coverage	Yes		1,354 (87.8)	
	No		189 (12.2)	
Parity	None		314 (20.4)	
	1–3		790 (51.2)	
	> 4		439 (28.4)	
Menopause	No		1,253 (18.8)	
	Yes		290 (81.2)	
Family history of BC	No		1,452 (94.1)	
	Yes		91 (5.9)	
Mass media exposure	No		311 (20.2)	
	Yes		1,232 (79.8)	
Perceived accessibility	No		1,014 (65.7)	
	Yes		529 (34.3)	
Knowledge of BC		0–22		15.07 ± 5.96
Health Belief and BC perceptions	Perceived susceptibility	1–5		2.93 ± 0.98
	Perceived benefits	1–5		3.59 ± 0.75
	Perceived barriers	1–5		2.82 ± 0.56
	Fatalism	1–11		6.38 ± 2.93
	Perceived treatment belief	1–5		3.44 ± 0.35
	Perceived stigma	1–5		3.89 ± 0.55
	Perceived fear	1–5		3.91 ± 0.69
	Cultural belief	0–16		7.06 ± 3.02
Distance to a health facility is a problem	No		897 (58.1)	
	Yes		646 (41.9)	
**Interpersonal factors**				
Friends’ recommendation	No		1,261 (81.7)	
	Yes		282 (18.3)	
Family recommendation	No		1,293 (83.8)	
	Yes		250 (16.2)	
Health providers Recommendations	No		1,201 (77.8)	
	Yes		342 (22.2)	
Social support		1–5		3.90 ± 0.80
**Contextual factors**				
Availability of BCS center/hospital	No		1,319 (85.5)	
	Yes		224 (14.5)	
Distance to a health facility	< 5 km		363 (23.5)	
	5–10 km		867 (56.2)	
	> 10 km		313 (20.3)	
Residence	Rural		1,020 (66.1)	
	Urban		523 (33.9)	
Sub-metropolitan Assembly	Tamale South		635 (41.1)	
	Tamale North		586 (38.0)	
	Tamale Central		322 (20.9)	

*BC *Breast cancer, *BCS *Breast cancer screening, *GHS *Ghana Cedi, *km *kilometers, *M *Mean, *SD *Standard deviation

### Association between CBE uptake and explanatory variables

Only 354 (23%) of participants reported having had CBE in the past year. From the bivariate analysis (Table 2) the following were positively associated with CBE uptake: 11 *intrapersonal* variables (educational status, employment status, monthly income level, mass media exposure, knowledge of BC, perceived susceptibility, perceived barriers, perceived stigma, fatalism, perceived fear, distance to a health facility a problem); all *interpersonal* variables (recommendations from friends, family, and healthcare providers, social support); and all *contextual* variables (availability of BC screening center/health facility, distance to a health facility, area of residence and sub-metropolitan assembly).

**Table 2:** Bivariate association between clinical breast examination and explanatory variables (*n* = 1,543)

Variables	Category	No CBE(1,189)	Yes CBE(354)	
		*n*(%)M ± SD	*n*(%)M ± SD	*P* value
**Intrapersonal factors**				
Age in years		40.58 ± 12.02	39.73 ± 10.22	0.228
Marital status	Never married/single	194(80.5)	47(20.50	
	Divorced/widowed	72(76.6)	22(23.4)	0.384
	Married/cohabitating	923(76.4)	285(23.6)	
Educational status	No education	542(84.2)	102(15.8)	
	Primary/junior high	329(83.9)	63(16.1)	< 0.001
	Secondary	244(77)	73(23)	
	Tertiary	74(39.0)	116(61.0)	
Religious status	No religion/Traditionalist	10(83.3)	2(16.7)	0.059
	Christian	105(63.6)	60(36.4)	
	Muslim	1074(78.6)	292(21.4)	
Employment status	No	801(82.8)	167(17.2)	
	Yes, Part-time	134(77.9)	38(22.1)	< 0.001
	Yes, Full-time	254(63.0)	149(37.0)	
Income level	Less 500 GHS	847(84.1)	160(15.9)	
	500–1000 GHS	253(73.3)	92(26.7)	< 0.001
	> 1000 GHS	89(46.6)	102(53.4)	
Health insurance coverage	Yes	1,035(76.5)	319(23.5)	0.121
	No	154(81.5)	35(18.5)	
Parity	None	248(79.0)	66(21.0)	0.657
	1–3	604(76.5)	186(23.5)	
	> 4	337(76.8)	102(23.2)	
Menopause	No	956 (76.3)	297(23.7)	0.140
	Yes	233(80.3	57(19.7)	
Family history of BC	No	1,121(77.2)	331(22.8)	0.585
	Yes	68(74.7)	23(25.3)	
Mass media exposure	No	263(84.6)	48(15.4)	< 0.001
	Yes	926(75.2)	306(24.8)	
Knowledge of BC		14.62 ± 6.21	16.57 ± 4.71	< 0.001
Health beliefs and BC perceptions	Perceived susceptibility	2.86 ± 0.98	3.17 ± 0.93	< 0.001
	Perceived benefits	3.59 ± 0.78	3.60 ± 0.65	0.797
	Perceived barriers	2.90 ± 0.51	2.55 ± 0.65	< 0.001
Fatalistic beliefs and perceptions of BC	Fatalism	6.47 ± 2.96	6.07 ± 0.2.83	0.024
	Perceived stigma	3.91 ± 0.53	3.84 ± 0.61	< 0.001
	Perceived fear	3.93 ± 0.67	3.81 ± 0.75	< 0.001
	Cultural beliefs	7.11 ± 3.04	6.86 ± 2.93	0.164
Distance to health facility is a problem	No	646(72.0)	251(28.0)	< 0.001
	Yes	543(84.1)	103(15.9)	< 0.001
**Interpersonal factors**				
Friends’ recommendation	No	1,055(83.7)	206(16.3)	< 0.001
	Yes	134(47.5)	148(52.5)	
Family recommendation	No	1,071(82.8)	222(17.2)	< 0.001
	Yes	118(47.2)	132(52.8)	
Social support		3.88 ± 0.78	3.98 ± 0.80	0.038
Health providers Recommendations	No	994(82.8)	207(17.2)	< 0.001
	Yes	195(57.0)	147(43.0)	
**Contextual factors**				
Availability of BCS center/hospital	No	1,080(81.9)	239(18.1)	< 0.001
	Yes	109(48.7)	115(51.3)	
Distance to a health facility	< 5 km	240(66.2)	123(33.9)	
	5–10 km	688(79.4)	179(20.6)	< 0.001
	> 10 km	261(83.4)	52(16.6)	
Residence	Rural	818(80.2)	202(19.8)	< 0.001
	Urban	371(70.9)	152(29.1)	
Sub-metropolitan Assembly	Tamale South	531(83.6)	104(16.4)	
	Tamale North	445 (75.9)	141(24.1)	< 0.001
	Tamale Central	213(66.2)	109(33.8)	

*BC *Breast cancer, *BCS *Breast cancer screening, *CBE *Clinical breast examination, *GHS *Ghana Cedi, *km *kilometers, *M *Mean, *SD *Standard deviation

With regard to educational status, women with tertiary education (61.0%) recorded the highest proportion of CBE uptake. Women who were fully employed (37.0%) had the highest proportion of CBE uptake, while the unemployed had the lowest proportion. Women with more than 1000 GHC monthly incomes (53.4%), a family history of BC (25.3%), and mass media exposure (24.8%) had the highest proportion of CBE uptake. In relation to women’s knowledge of BC, those who utilized CBE had the highest mean knowledge score (M = 16.57 ± SD = 4.71) than those who did not utilize CBE. Women who utilized CBE had the highest mean score on perceived susceptibility (M = 3.17 ± SD = 0.93), and the lowest mean score in perceived barriers (M = 2.55 ± SD = 0.65), fatalism (M = 6.07 ± SD = 0.2.83), perceived stigma (M = 3.84 ± SD = 0.61), perceived fear (M = 3.81 ± SD = 0.75), and cultural beliefs (M = 6.86 ± SD = 2.93), compared to those who did not utilize CBE. Women who resided in communities where health facilities/screening centers were available (51.3%) and resided in urban areas (29.1%) had the highest proportion of CBE uptake than their counterparts (Table 2).

### Fixed and random effects (measures of variations) results of clinical breast examination

From Table 3, the random intercept model showed a significant variation in the likelihood of CBE among women in the Tamale metropolis across the PSUs clustering (σ2 = 1.40 95%CI = 0.21,0.77). Model 0 revealed that 10.9% of the variation in CBE uptake in the Tamale metropolis was attributed to the variation between-cluster characteristics, evidenced by the ICC of 0.1098928. The variation between clusters reduced drastically to about 5.6% in Model I which included only the intrapersonal factors. In Model II, which adjusted for the interpersonal factors, the ICC decreased to about 4.5%. After adjusting for the contextual factors in Model III, there was a further slight decline in the ICC to about 4.1%. The ICC values showed a successive reduction from Model 0 to Model III, which meant that there was a significant improvement in each model over the prior model affirming the goodness of the final model (Model III). To further emphasize the goodness of the model, the log-likelihood ratio successively increased from the random intercept model (−796.85) to the final model (−630.95). The final model showed that a 4.1% variation in CBE uptake among women may be attributed to contextual level factors. In reporting the results, the best-fit model was selected based on the highest loglikelihood value among the models. Therefore, the final model (Model III) consisting of all the independent variables was chosen to present the factors that were associated with CBE uptake.

**Table 3:** Multilevel analysis of factors associated with the utilization of clinical breast examination

Variables	Categories	Model 0	Model I	Model II	Model III
			aOR[95%CI]	aOR[95%CI]	aOR[95%CI]
**Intrapersonal factors**					
Age in years	25-39years		1	1	1
	40-55years		1.14[0.84,1.54]	1.11[0.81,1.51]	1.16[0.85,1.60]
	55–75 years		0.74[0.44,1.23]	0.65[0.38,1.12]	0.69[0.40,1.18]
Educational status	No education		1	1	1
	Primary/Junior high		0.87[0.59,1.27]	0.86[0.58,1.28]	0.88[0.59,1.32]
	Secondary		1.16[0.78,1.72]	1.02[0.68,1.54]	1.00[0.66,1.52]
	Tertiary		3.67^***^[2.23,6.02]	2.43^***^[1.45,4.07]	2.53^***^[1.49,4.29]
Employment status	No		1	1	1
	Yes, Part-time		0.87[0.52,1.47]	0.75[0.43,1.28]	0.77[0.44,1.34]
	Yes, Full-time		1.26[0.83,1.93]	1.16[0.75,1.77]	1.29[0.83,1.99]
Income level	Less than 500 GHS		1	1	1
	500–1000 GHS		1.051[0.69,1.61]	1.107[0.72,1.71]	0.988[0.63,1.55]
	> 1000 GHS		1.320[0.74,2.34]	1.441[0.80,2.59]	1.249[0.69,2.27]
Mass media exposure	No		1	1	1
	Yes		1.13[0.77,1.66]	0.99[0.66,1.47]	1.00[0.67,1.51]
Knowledge of BC			1.03^*^[1.00,1.06]	1.03[1.00,1.06]	1.04^*^[1.00,1.07]
Health Belief and BC perceptions	Perceived susceptibility		1.26^**^[1.08,1.46]	1.25^**^[1.07,1.47]	1.25^**^[1.06,1.47]
	Perceived barriers		0.44^***^[0.33,0.58]	0.54^***^[0.41,0.72]	0.54^***^[0.41,0.73]
	Fatalism		0.95^*^[0.90,1.00]	0.95[0.90,1.01]	0.95[0.90,1.01]
	Perceived fear		0.76^*^[0.63,0.96]	0.68^***^[0.54,0.86]	0.72^**^[0.57,0.91]
Distance to a health facility is a problem	No		1	1	1
	Yes		0.53^***^[0.38,0.73]	0.48^***^[0.34,0.68]	0.56^**^[0.39,0.80]
Healthcare costs a problem	No		1	1	1
	Yes		1.65^**^[1.16,2.35]	1.42[0.98,2.06]	1.26[0.86,1.84]
**Interpersonal factors**					
Friends’ recommendation	No			1	1
	Yes			2.11^**^[1.33,3.36]	1.99^**^[1.25,3.18]
Family recommendation	No			1	1
	Yes			1.69^*^[1.05,2.73]	1.60[0.99,2.58]
Health providers recommendations	No			1	1
	Yes			1.76^**^[1.15,2.68]	1.55^*^[1.00,2.39]
Social support				0.89[0.72,1.11]	0.86[0.69,1.06]
**Contextual factors**					
Availability of BCS center/hospital	No				1
	Yes				2.14^***^[1.43,3.21]
Distance to a health facility	< 5 km				1
	5–10 km				0.66[0.39,1.13]
	> 10 km				0.51[0.26,1.02]
Residence	Rural				1
	Urban				1.72^*^[1.10,2.69]
Sub-metropolitan Assembly	Tamale South				1
	Tamale North				0.69[0.45,1.07]
	Tamale Central				0.82[0.47,1.40]
Fixed effects results	PSU variance	1.40[0.21,0.77]	1.21[1.01,1.48]	1.16[0.98,1.39]	1.15[0.97,1.37]
	ICC	0.1098928	0.0566755	0.0446593	0.0412404
	Wald χ2	Reference	185.98***	228.23***	238.63***
Model fitness	Log-likelihood	−796.85	−681.18	−642.81	−630.95
Number of clusters		48	48	48	48
*N*		1543	1543	1543	1543

^*^p < 0.05; ^**^p < 0.01; ^***^p < 0.001; χ2 = Chi-square; 1 = reference category; *aOR *adjusted odds ratio, *BC *Breast cancer, *BCS *Breast cancer screening, *CI *Confidence intervals, *GHS *Ghana Cedi, *ICC *Intra-class correlation, *km *kilometers, *PSU *Primary sampling unit

### Factors associated with the utilization of clinical breast examination

From the fixed effects results, women with tertiary education (aOR = 2.53, 95%CI = 1.49–4.29) were more likely to have CBE compared to those without formal education. Women in communities with available BC screening/health facilities (aOR = 2.14, 95%CI = 1.43–3.21) and those who resided in urban areas (aOR = 1.72, 95%CI = 1.10–2.69) were more likely to utilize CBE. Women who received recommendations from friends (aOR = 1.99, 95%CI = 1.25–3.18) and healthcare providers (aOR = 1.55, 95%CI = 1.00-2.39) were more likely to utilize CBE than those who did not receive any recommendation. Women with increased knowledge of BC were more likely to undergo CBE (aOR = 1.04, 95%CI = 1.00-1.07). The higher the women’s perceived susceptibility to BC, the higher their likelihood of having CBE (aOR = 1.25, 95%CI = 1.06–1.47). Women with increased perceived barriers to BC screening (aOR = 0.54, 95%CI = 0.41–0.73) and higher perceived fear of BC (aOR = 0.72, 95%CI = 0.57–0.91) were less likely to utilize CBE. Women who perceived the distance to health facilities to be a problem were less likely to utilize CBE (aOR = 0.56, 95%CI = 0.39–0.80). (Table 3).

## Discussion

This was the first community-based study employing the ecological model to examine intrapersonal, interpersonal, and community-level factors influencing CBE uptake. In the wake of rising BC incidence in Ghana, this study aimed to provide a comprehensive picture of CBE uptake among women in the Northern Region of Ghana. The present study found that only 23% of women utilized CBE, which is higher than the 14.4% reported by a recent study in the Upper West Region of Ghana [6]. A plausible explanation for the current finding could be attributed to the BC campaigns initiated by the Oncology unit of the Tamale Teaching Hospital in the past five years, where advocacy programs and outreach community-based CBE for remote communities. Also, our finding is higher than a recent study conducted in Lesotho, where 9.73% of women of reproductive age utilized CBE [25], which may be due to inadequate health infrastructure and services, fewer healthcare professionals, and a lack of established BC screening programs and treatment facilities in Lesotho. Though efforts are made to ensure early detection of BC among women within the region and Ghana at large, more extensive public awareness campaigns on BC are required, specifically in deprived areas where healthcare access is poor. Despite the majority of the women having health insurance, the CBE rates are low compared to a study conducted in the Northern Region of Ghana, with 38% [13]. The difference between our findings and Gyedu et al. [13] may be due to the differences in the study populations. The earlier study [13] sampled women primarily from Christian and Muslim religious organizations, many of which offer health promotion initiatives, including free BC screening for their members, possibly leading to a higher screening rate than the current study. In contrast, the majority of participants in our study were rural residents (66.1%), where access to healthcare services, including CBE, is often limited. Notably, over 90% of women in Gyedu et al. [13] study, lived in urban areas, compared to only 33.9% in our sample. The disparity in access to health education and screening services between urban and rural settings likely contributed to the lower CBE rate observed in our study. Intensified, community-driven public health education and outreach, especially in underprivileged areas is urgently needed to increase CBE uptake in the Northern Region of Ghana. Using current health infrastructure, like the Tamale Teaching Hospital’s advocacy activities, together with focused awareness campaigns and local health worker training, might help close knowledge gaps and foster early detection practices. Furthermore, including CBE promotion into regular basic healthcare services, particularly when mammography is lacking, will be crucial in resolving inequalities and improving BC screening rates among women.

This study found that women who attained tertiary education were more likely to utilize CBE than those with no formal education. This finding disagrees with a recent study in the Upper West Region of Ghana, where women with tertiary education were less likely to utilize breast self-examination, CBE, and MMG [6]. Wuur et al. [6] explained that the women might have been overwhelmed with work schedules and could not book appointments or attend BC screening. Also, educational status has a significant positive relationship with the level of health literacy [26]. It is possible that highly educated women would have a better comprehension of health check-up concepts, as well as a belief in detecting asymptomatic diseases early [27]. Furthermore, it is believed that educated women may also have health knowledge about BC and understand the ripple effect if not detected early, and therefore take proactive preventive measures to overcome any barrier to participating in CBE [28]. Therefore, efforts such as public BC campaigns and providing access to CBE are essential to target Ghanaian women who lack formal education. Due to the culturally diverse nature of the Ghanaian communities and the different dialects of communication, it is important that BC education programs should be culturally tailored for such women.

The significant positive association between BC knowledge and CBE uptake in this study aligns with studies conducted in Vietnam [29], Malaysia [30], and Sri Lanka [31]. A plausible explanation is that knowledge of BC contributes to positive preventive measures taken by women in order to prevent the risk of developing BC, hence influencing women to utilize CBE. However, we acknowledge that the relationship may be bidirectional. While higher knowledge of BC can promote CBE uptake, it is also possible that undergoing CBE provides an opportunity for women to receive education on breast health, thereby improving their knowledge. This reciprocal relationship underscores the value of integrating education into screening programs. Indeed, an educational intervention study on early detection of BC in Sri Lanka found that the post-intervention group had higher knowledge and a higher CBE uptake rate compared with the pre-intervention group [31]. We recommend implementing educational interventions alongside screening programs to improve both knowledge and participation in early detection practices.

Consistent with several other cross-sectional studies conducted in the Maldives [30], Iran [32], and Vietnam [29], women with higher perceptions of susceptibility to BC were more likely to utilize CBE than those with lower susceptibility. On the other hand, this study revealed that women who had fewer perceived barriers to screening were more likely to utilize CBE, which is consistent with previous studies [29, 30, 32, 33]. Therefore, interventions that seek to overcome these perceived barriers to BC screening should be adopted and implemented to promote screening.

The present study reveals the significant positive effect of friends’ and healthcare professionals’ recommendations on CBE uptake. This finding underscores the crucial role healthcare professionals play in influencing the health behavior of women, along with the significant trust in healthcare professionals’ recommendations regarding health decisions. Since the study findings suggest the significance of health professional recommendations in promoting BC screening, integrating BC screening programs with existing health programs and services, such as reproductive health programs, maternal and child health services, or general health check-ups, would be imperative.

This finding is congruent with a previous nationwide study in Kenya, which reported that women in urban settlements were more likely to undergo CBE because screening centers were more available [34]. However, it is inconsistent with recent evidence in Lesotho, where it was found that the area of residence (urban vs. rural) did not significantly influence CBE uptake [25]. The present study’s findings might be attributed to the fact that rural communities in developing countries often lack health infrastructure, such as hospitals and other health facilities, but these are often made available in urban settings [34]. It has been stressed that if women do not have to travel long distances to access CBE, they would be motivated to undergo CBE due to proximity [35]. Thus, CBE uptake among Ghanaian women could be improved if barriers such as unavailability and inaccessibility of healthcare facilities are addressed in rural areas. Due to the unavailability of screening facilities, coupled with the majority of Ghanaian women presenting with advanced BC cases in health facilities, the guidelines from the Breast Health Global Initiative, which suggest the expansion of early detection services with CBE, should be adopted and intensified [35, 36]. Due to the limited access or disproportionate distribution of health facilities between urban and rural communities, this study recommends that CBE should be made available in non-hospital community-level health facilities such as public health centers, and priorities should be given to training community-level health personnel to undertake this task because these facilities are widespread and easier to access than hospitals. This approach would provide expanded services to a greater proportion of the Ghanaian population of women and enhance CBE uptake.

### Limitations of the study

This study used a cross-sectional design and therefore could not draw a causal inference; hence, the findings should be interpreted with caution. Even though the study was conducted across 30 communities with about 48 clusters, these findings cannot be generalized to the population of Ghanaian women due to regional and contextual differences. Although this study had limitations, the use of a larger sample with a robust sampling method and validated tools to collect data from women and the use of a multilevel method of analysis gives credence to the study findings.

## Conclusion

The study revealed a very low rate, and multilevel factors associated with CBE uptake. The study findings signify that the interplay of individual, interpersonal, and community-level factors influencing women’s BC screening behavior requires a multifactorial systematic approach to increase CBE uptake among Ghanaian women. We recommend a comprehensive community-based education program about BC targeted at raising awareness of breast health and improving the knowledge of the disease, together with the benefits of CBE uptake.

## Supplementary Information

Supplementary Material 1. The study questionnaire has been attached as a supplementary file.

## Data Availability

The data for this study is available to the corresponding author based on a reasonable request.

## Abbreviations

aOR: Adjusted Odds Ratio
BC: Breast Cancer
BCAM: Breast Cancer Awareness Measure
CBE: Clinical Breast Examination
CI: Confidence Interval
GDHS: Ghana Demographic and Health Survey
ICC: Intra-Class Correlation
MMG: Mammography
MOS: Medical Outcome Study
PSUs: Primary Sample Units
SD: Standard Deviation
VIF: Variance Inflation Factor
