# Supplementary material for: Multilevel factors associated with clinical breast examination uptake among women in the Northern Region of Ghana: a cross-sectional survey
Source: BMC Cancer. 2025 Oct 1;25:1476. doi: 10.1186/s12885-025-14550-w (PMC12487212; doi:10.1186/s12885-025-14550-w)
Supplement: Supplementary file 1 — Supplementary Material 1. The study questionnaire has been attached as a supplementary file. [file 12885_2025_14550_MOESM1_ESM.docx]

**Supplementary file 1**

**Questionnaire**

**Informed consent**

Hello. My name is __________. I am working with the Ministry of Health/Ghana Health Service. We are conducting a survey about breast cancer and breast cancer screening and mammography intention all over the Tamale metropolis.

The information we collect will be for academic purposes and to help the government to plan health services regarding breast cancer screening. Your household was selected for the survey. The questions usually take about 30 to 45 minutes. All of the answers you give will be confidential and will not be shared with anyone other than members of our survey team. You don't have to be in the survey, but we hope you will agree to answer the questions since your views are important. If I ask you any question you don't want to answer, just let me know and I will go on to the next question or you can stop the interview at any time. In case you need more information about the survey, you may contact the person listed on the card that has already been given to your household.

Do you have any questions? May I begin the survey now?

**For Research Assistant**

1. Indicate the cluster number given provided__________________
2. Sub-metropolitan assembly
3. Tamale North
4. Tamale South
5. Tamale Central

**Outcome measures**

1. Have you done breast self-examination in the past year?
2. Never
3. Sometimes
4. Often
5. Regularly
6. Have you had a breast examination by a health professional in the past year?
7. No
8. Yes
9. How many mammograms have you had for breast cancer screening in the last two years?
10. None
11. Once
12. Twice
13. How much do you agree with the statement? - “I intend to have mammography (X-ray of the breasts to screen for cancer) within the next two years.”
14. Strongly disagree
15. Disagree
16. Neutral
17. Agree
18. Strongly agree

**Individual level measures**

***Knowledge of risk, protective factors, and good practice of breast cancer***

| **No** | **Items** | **1** | **2** | **3** | **4** | **5** |
| --- | --- | --- | --- | --- | --- | --- |
|  | **How much do you agree with the following as breast cancer risk factors?** |  |  |  |  |  |
|  | Is alcohol intake a risk factor? |  |  |  |  |  |
|  | Is Smoking a risk factor? |  |  |  |  |  |
|  | Is Nulliparity/late maternity a risk factor? |  |  |  |  |  |
|  | Is Physical inactivity and obesity a risk factor? |  |  |  |  |  |
|  | **How much do you agree with the following as breast cancer protective measures?** |  |  |  |  |  |
|  | Does Breastfeeding protect you from breast cancer? |  |  |  |  |  |
|  | Does Early maternity (first child before age 30) protect you from breast cancer? |  |  |  |  |  |
|  | Does a balanced diet and lifestyle protect you from breast cancer? |  |  |  |  |  |
|  | **How much do you agree with the following as breast cancer protective measures?** |  |  |  |  |  |
|  | Is breast self-examination a good practice? |  |  |  |  |  |
|  | Is Clinical breast examination a good practice? |  |  |  |  |  |
|  | Is Mammography a good practice? |  |  |  |  |  |
|  | Who is most likely to get breast cancer? Please select only one answer.  a) A 30-year-old woman  b) A 50-year-old woman  c) A 70-year-old woman  d) A woman of any age |  |  |  |  |  |

Legend: 1=strongly disagree, 2= disagree, 3=neutral, 4=agree, 5=strongly agree

**Knowledge of warning signs**

|  | **Items**  **Are the following signs warning signs of breast cancer?** | **Yes** | **No** | **Don’t know** |
| --- | --- | --- | --- | --- |
|  | Change in the position of the nipple |  |  |  |
|  | Nipples being pulled inward |  |  |  |
|  | Pain in one of your breasts or armpits |  |  |  |
|  | Puckering or dimpling of your breast skin |  |  |  |
|  | Discharge or bleeding from your nipple |  |  |  |
|  | A lump or thickening in your breast |  |  |  |
|  | Nipple rash |  |  |  |
|  | ‘Redness’ of your breast skin |  |  |  |
|  | A lump or thickening under your armpit |  |  |  |
|  | Change in the size of your breast or nipple |  |  |  |
|  | Change in the shape of your breast or nipple |  |  |  |

**Perceived susceptibility, benefits, and barriers (CHBMS_BC) scale**

| **NO** |  | **How much do you agree with the following about breast cancer and screening?** | **1** | **2** | **3** | **4** | **5** |
| --- | --- | --- | --- | --- | --- | --- | --- |
|  |  | ***Susceptibility*** |  |  |  |  |  |
|  | 1 | It is likely that I will get breast cancer |  |  |  |  |  |
|  | 2 | My chances of getting breast cancer in the next few years are high |  |  |  |  |  |
|  | 3 | I feel I will get breast cancer sometime during my life |  |  |  |  |  |
|  |  | ***Benefits*** |  |  |  |  |  |
|  | 1 | If I get screened for breast cancer and nothing is found, I don’t need to worry as much about breast cancer |  |  |  |  |  |
|  | 2 | Having breast cancer screening will help me find breast lumps early |  |  |  |  |  |
|  | 3 | Having breast cancer screening is the best way for me to find a very small lump |  |  |  |  |  |
|  | 4 | Having breast cancer screening will decrease my chances of dying from breast cancer |  |  |  |  |  |
|  |  | ***Barriers*** |  |  |  |  |  |
|  | 1 | I am afraid to have breast cancer screening because I might find out something is wrong |  |  |  |  |  |
|  | 2 | I am afraid to have breast cancer screening because I don’t understand what will be done |  |  |  |  |  |
|  | 3 | I don’t know how to go about getting breast cancer screening |  |  |  |  |  |
|  | 4 | Having breast cancer screening is too embarrassing |  |  |  |  |  |
|  | 5 | Having breast cancer screening takes too much time |  |  |  |  |  |
|  | 6 | Having breast cancer screening is too painful |  |  |  |  |  |
|  | 7 | People doing breast cancer screening are rough to women. |  |  |  |  |  |
|  | 8 | Having breast cancer screening exposes me to unnecessary radiation |  |  |  |  |  |
|  | 9 | I cannot remember to go to the doctor to get breast cancer screening |  |  |  |  |  |
|  | 10 | I have other problems more important than getting breast cancer screening |  |  |  |  |  |
|  | 11 | I am not the right age to need a routine breast cancer screening |  |  |  |  |  |
|  | 12 | I cannot afford to get breast cancer screening |  |  |  |  |  |
|  | 13 | I don’t have the encouragement I need from my close relatives to attend breast cancer screening |  |  |  |  |  |
|  | 14 | I am afraid that a male doctor will carry out the breast cancer screening |  |  |  |  |  |

**Legend: 1=strongly disagree, 2= disagree, 3=neutral, 4=agree, 5=strongly agree**

***Fatalism scale***

**The following questions are concerned with the health issues of women your age. In this section, I will read each statement to you. I will then repeat the statement and ask for a YES, NO or Don’t Know answer. Please answer the following questions based on what you think. Remember that there are no right or wrong answers. Do you have any questions before starting this section?**

| **N0** |  | **Items** | **Yes** | **No** | **Don’t know** |
| --- | --- | --- | --- | --- | --- |
|  |  | **I believe…** |  |  |  |
|  | 1 | if someone gets breast cancer it was meant to be |  |  |  |
|  | 2 | if someone gets breast cancer, they will get it no matter what they do |  |  |  |
|  | 3 | if someone is meant to have breast cancer, they will have breast cancer. |  |  |  |
|  | 4 | if someone was meant to have breast cancer it doesn’t matter what the doctor tells them to do, they will get breast cancer anyway |  |  |  |
|  | 5 | if someone gets breast cancer it is part of God’s plan. |  |  |  |
|  | 6 | if someone gets breast cancer it doesn’t matter when they find out about it, they will still die. |  |  |  |
|  | 7 | if someone has breast cancer it is already too late to do anything about it. |  |  |  |
|  | 8 | if someone gets breast cancer their time to die is near |  |  |  |
|  | 9 | if someone gets breast cancer that’s the way they were meant to die |  |  |  |
|  | 10 | if someone gets breast cancer a lot of different treatments won’t make any difference. |  |  |  |
|  | 11 | breast cancer kills most people who get it. |  |  |  |

***Breast Cancer Perception Scale (perceived treatment belief, stigma, and fear) scale***

| **No** |  | **How much do you agree with the following as breast cancer perceptions?** | **1** | **2** | **3** | **4** | **5** |
| --- | --- | --- | --- | --- | --- | --- | --- |
|  |  | ***Perceived treatment belief*** |  |  |  |  |  |
|  | 5 | It is important for early diagnosis and treatment to attend screenings regularly |  |  |  |  |  |
|  | 6 | Early diagnosis of breast cancer increases the chances of recovery |  |  |  |  |  |
|  | 7 | *Breast cancer is a treatable disease |  |  |  |  |  |
|  | 8 | Clinical breast examination is important for early diagnosis and treatment |  |  |  |  |  |
|  | 9 | *Breast cancer treatment does not change the outcome |  |  |  |  |  |
|  |  | ***Perceived stigma*** |  |  |  |  |  |
|  | 14 | Women with breast cancer experience problems in their sexual lives |  |  |  |  |  |
|  | 15 | Women with breast cancer cannot take care of their children |  |  |  |  |  |
|  | 16 | Women with breast cancer experience problems in their marriages |  |  |  |  |  |
|  | 17 | Breast cancer treatment makes a woman less beautiful |  |  |  |  |  |
|  |  | ***Perceived fear*** |  |  |  |  |  |
|  | 18 | It scares me to think of breast cancer |  |  |  |  |  |
|  | 19 | I feel uncomfortable when I think of breast cancer |  |  |  |  |  |
|  | 20 | It makes me feel uneasy to think about the breast cancer treatment process |  |  |  |  |  |
|  | 21 | The thought of having breast cancer worries me |  |  |  |  |  |

**Legend: 1=strongly disagree, 2= disagree, 3=neutral, 4=agree, 5=strongly agree**

**We have covered half of the survey. If you need a short rest, please let me know.**

***Cultural belief scale:*** People have different opinions about breast cancer symptoms and its treatment. We are interested in your opinions about the following statements. Please indicate if you think these statements are TRUE or FALSE.

|  |  | **Items** | **True** | **False** |
| --- | --- | --- | --- | --- |
|  |  | ***Breast lump characteristics*** |  |  |
|  | 1 | If a breast lump is not painful, it is not cancer |  |  |
|  | 2 | If a breast lump does not get bigger, it is not cancer |  |  |
|  | 3 | If a breast lump is touched/pressed often, it will turn out to be breast cancer |  |  |
|  | 4 | Women with large breasts are more likely to get breast cancer than women with small breasts. |  |  |
|  |  | ***Self-help techniques*** |  |  |
|  | 5 | The more you worry about breast cancer, the more likely you will get it |  |  |
|  | 6 | If you take good care of yourself, you won’t get breast cancer. |  |  |
|  | 12 | If you don’t have breast cancer in your family, you don’t need to get mammograms. |  |  |
|  | 13 | If you have a breast lump, a “natural” remedy can help to get rid of it. |  |  |
|  | 9 | Clinical breast examination/breast self-examination/ mammography causes breast cancer |  |  |
|  |  | ***Faith-based beliefs*** |  |  |
|  | 7 | Faith in God can protect you from breast cancer. |  |  |
|  | 10 | If you pray enough, sometimes breast lumps will disappear. |  |  |
|  | 14 | If a woman has enough faith in God, she won’t need treatment for breast cancer |  |  |
|  |  | ***Futility of treatment*** |  |  |
|  | 8 | You only need to get breast cancer screening if you find a problem in your breast. |  |  |
|  | 11 | If breast cancer is cut open in surgery, it will grow faster |  |  |
|  | 15 | If a woman is poor, she will not get cured from cancer because she won’t get the best treatment |  |  |
|  | 16 | If breast cancer is treated correctly, it can be cured |  |  |
|  | 17 | It doesn’t really matter if you get treated for breast cancer, because if you get cancer, it will kill you sooner or later. |  |  |

**Interpersonal level factors**

1. Have you ever received recommendations from friends on breast self-examination?
2. Yes
3. No
4. Have you ever received recommendations from family members on breast self-examination?
5. Yes
6. No
7. Have you ever received recommendations from friends for having your breast screened by a health professional?
8. Yes
9. No
10. Have you ever received recommendations from family members to have your breast screened by a health professional?
11. Yes
12. No
13. Have you ever received recommendations from friends to undergo mammography?
14. Yes
15. No
16. Have you ever received recommendations from family members to undergo mammography?
17. Yes
18. No
19. Have you ever received recommendations from health professionals to practice breast self-examination?
20. Yes
21. No
22. Don’t know
23. Have you ever received recommendations from health care professionals to receive clinical breast examinations?
24. Yes
25. No
26. Don’t know
27. Have you ever received recommendations from health care professionals to have mammography?
28. Yes
29. No
30. Don’t know

**Social support**

People sometimes look to others for companionship, assistance, or other types of support. How often is each of the following kinds of support available to you if you need it? Choose one number from each line.

| **No** |  | **Items** | **1** | **2** | **3** | **4** | **5** |
| --- | --- | --- | --- | --- | --- | --- | --- |
|  | 1 | Someone to help you if you were confined to bed |  |  |  |  |  |
|  | 2 | Someone you can count on to listen to you when you need to talk |  |  |  |  |  |
|  | 3 | Someone to give you good advice about a crisis |  |  |  |  |  |
|  | 4 | Someone to take you to the doctor if you needed it |  |  |  |  |  |
|  | 5 | Someone who shows you love and affection |  |  |  |  |  |
|  | 6 | Someone to have a good time with |  |  |  |  |  |
|  | 7 | Someone to give you information to help you understand a situation |  |  |  |  |  |
|  | 8 | Someone to confide in or talk to about yourself or your problems |  |  |  |  |  |
|  | 9 | Someone who hugs you |  |  |  |  |  |
|  | 10 | Someone to get together with for relaxation |  |  |  |  |  |
|  | 11 | Someone to prepare your meals if you were unable to do it yourself |  |  |  |  |  |
|  | 12 | Someone whose advice you really want |  |  |  |  |  |
|  | 13 | Someone to do things with to help you get your mind off things |  |  |  |  |  |
|  | 14 | Someone to help with daily chores if you were sick |  |  |  |  |  |
|  | 15 | Someone to share your most private worries and fears with |  |  |  |  |  |
|  | 16 | Someone to turn to for suggestions about how to deal with a personal problem |  |  |  |  |  |
|  | 17 | Someone to do something enjoyable with |  |  |  |  |  |
|  | 18 | Someone who understands your problems |  |  |  |  |  |
|  | 19 | Someone to love and make you feel wanted |  |  |  |  |  |

**Legend: 1=**None of the time, 2=A little of the time, 3=Some of the time, 4=Most of the time, 5=All of the time

1. Is there any health facility that offers breast cancer screening within the community (RA confirms)?
2. Yes
3. No
4. Don’t Know
5. Do the regional health facilities organize public health campaigns on breast cancer screening?
6. Yes
7. No
8. Don’t Know
9. Is there any available screening facility within the community? (RA confirm)
10. Yes
11. No
12. Don’t Know
13. Is the cost of seeking health care a problem for breast cancer screening?
14. Yes
15. No
16. Don’t know

**Sociodemographic characteristics**

1. What is your age in years ________________________
2. Your marital status
3. Never married/single
4. Cohabitation
5. Divorced/widowed/separated
6. Married
7. What is your level of last completed education?
8. No education
9. Primary/Junior high
10. Secondary
11. Tertiary
12. Which religion do you belong to?
13. No religion
14. Traditionalist
15. Christian
16. Muslim
17. Others
18. Are you currently employed?
19. Yes, Part-time
20. Yes, Full time
21. No
22. What is your income level per month?
23. Less 500 GHC
24. 500-1000 GHC
25. >1000 GHC
26. Are you covered by the national health insurance?
27. Yes
28. No
29. How many children have you given birth to?
30. None
31. 1-3
32. ≥4
33. Have you passed menopause (more than 12 months since your last menstrual period)?
34. Yes
35. No
36. Have any of your family members ever been diagnosed with breast cancer?
37. Yes
38. No
39. If Yes in the above question Who? ____________________
40. How frequently do you read the newspaper or magazine?
41. Not at all
42. Sometimes
43. Almost everyday
44. How frequently do you listen to the radio?
45. Not at all
46. Sometimes
47. Almost everyday
48. How frequently do you watch television?
49. Not at all
50. Sometimes
51. Almost everyday
52. Type of place of residence
53. Urban
54. Rural
55. Do you have access to breast cancer screening centers?
56. Yes
57. No
58. Is the distance to the health facility a problem?
59. Yes
60. No
61. How far is the distance to the breast cancer screening center (hospital)? (Using GPS coordinates) ______________Kilometers.

**This is the end of the survey.**

**Thank you again for your time.**
